# Mass Treatment with Azithromycin for Trachoma Control: Participation Clusters in Households

**DOI:** 10.1371/journal.pntd.0000838

**Published:** 2010-10-05

**Authors:** Elizabeth N. Ssemanda, Beatriz Munoz, Emma M. Harding-Esch, Tansy Edwards, Harran Mkocha, Robin L. Bailey, Ansumana Sillah, Dianne Stare, David C. W. Mabey, Sheila K. West, On behalf of PRET Project Team

**Affiliations:** 1 Dana Center for Preventive Ophthalmology, Wilmer Eye Institute, Johns Hopkins University, Baltimore, Maryland, United States of America; 2 London School of Hygiene and Tropical Medicine, London, United Kingdom; 3 Kongwa Trachoma Project, Kongwa, Tanzania; 4 National Eye Care Programme, Gambian Department of State for Health and Social Welfare, Ministry of Health, Banjul, The Gambia; University of Cambridge, United Kingdom

## Abstract

**Background:**

Mass treatment to trachoma endemic communities is a critical part of the World Health Organization SAFE strategy. However, non-participation may not be at random, affecting coverage surveys and effectiveness if infection is differential.

**Methodology/Principal Findings:**

As part of the Partnership for Rapid Elimination of Trachoma (PRET), 32 communities in Tanzania, and 48 in The Gambia had a detailed census taken followed by mass treatment with azithromycin. The target coverage in each community was >80% of children ages <10 years. Community treatment assistants observed treatment and recorded compliance, thus coverage at the community, household, and individual level could be determined. Within each community, we determined the actual proportions of households where all, some, or none of the children were treated. Assuming the coverage in children <10 years of the community was as observed and non-participation was at random, we did 500 simulations to derive expected proportions of households where all, some, or none of the children were treated. Clustering of household treatment was detected comparing greater-than-expected proportions of households where none or all of children were treated, and the intraclass correlation (ICC) was calculated. Tanzanian and Gambian mass treatment coverages for children <10 years of age ranged from 82–100% and 62–99%, respectively. Clustering of households where all children were treated or no children were treated was greater than expected. Compared to model simulations, all Tanzanian communities and 44 of 48 (91.7%) Gambian communities had significantly higher proportions of households where all children were treated. Furthermore, 30 of 32 (93.8%) Tanzanian communities and 34 of 48 (70.8%) Gambian communities had a significantly elevated proportion of households compared to the expected proportion where no children were treated. The ICC for Tanzania was 0.77 (95% CI 0.74–0.81) and for The Gambia was 0.55 (95% CI 0.51–0.59).

**Conclusions/Significance:**

In programs aiming for high coverage, complete compliance or non-compliance with mass treatment clusters within households. Non-compliance cannot be assumed to be at random.

## Author Summary

Trachoma, an infectious disease, continues to cause blindness. A great deal of the trachoma burden is concentrated in developing countries. The World Health Organization recommends mass treatment for entire communities in trachoma-endemic regions. In 32 Tanzanian and 48 Gambian communities with trachoma, mass treatment was directly observed following a census. Community coverage was mostly greater than 80%. Larger-than-expected proportions of households where all children were treated and where none of the children were treated were found in each country. Household clustering of treatment was higher in Tanzania compared to The Gambia. However, children who were not treated were not more likely to be infected compared to children who were treated. We found that treatment and non-treatment within communities does not occur at random but rather clusters within households. These findings impact the design of future coverage surveys and suggest that further research evaluate factors that are associated with familial non-compliance.

## Introduction

Trachoma is the leading infectious cause of blindness [1]. As the most common ocular neglected tropical disease, active trachoma is estimated to affect 40.6 million people worldwide, and another 8.2 million experience visual impairment or blindness [2]. Trachoma is largely confined to regions of extreme poverty [3].The World Health Organization (WHO) African region contains more than two thirds of all active trachoma cases and approximately 47% of all trichiasis cases [2].

The WHO recommends azithromycin mass drug administration as a key part of the Surgery, Antibiotics, Face-washing, Environmental change (SAFE) strategy for eliminating trachoma. The WHO advocates a treatment coverage goal of at least 80% to be effective [4]. Although evidence is needed to determine the impact of coverage at different thresholds, national trachoma control programs need to be able to measure non-participation in order to meet distribution targets. Because active trachoma and infection largely reside in preschool age children within communities, antibiotic treatment should particularly target this group for maximal effectiveness [5].

Treatment coverage surveys often carry an implicit assumption that missing treatment occurs at random. There are no data to support this conjecture, and this is reason for concern. There are ample data that trachoma clusters in families and in neighborhoods [6]–[10], and that transmission within households and across households does occur [10], [11]. If treatment also tends to cluster, and is differential by infection status, then even the effect of high coverage may be compromised. Moreover, any treatment clustering will affect the precision of coverage estimates, and how one designs coverage surveys.

We examined the clustering of treatment at the household level using data at baseline from 32 communities in central Tanzania and 48 communities in The Gambia who are enrolled in the three-year Partnership for Rapid Elimination of Trachoma (PRET) project.

## Methods

### Ethics statement

The study was approved by the Johns Hopkins Medical Institutional Review Board, the Tanzanian National Institute for Medical Research, the London School of Hygiene and Tropical Medicine Ethical Review Board, and The Gambia Government/Medical Research Council Joint Ethics Committee.

All individual participants for this study provided consent. All adults provided informed written consent in both The Gambia and Tanzania.

### Populations

The study was conducted in 32, geographically distinct, communities within the Kongwa district of Tanzania. Communities were eligible if they were located in the Kongwa district, the community leadership gave consent, and the estimated community prevalence of trachoma was greater than or equal to 20% and less than 50% in preschool age children. We excluded communities where the estimated population was greater than 5,000 persons. In Tanzania, a household was defined as persons who used a unique doorway to sleeping quarters.

In The Gambia, Census Enumeration Areas (EAs) were used as communities because villages (collection of households with a distinct name) varied so much in size. The estimated prevalence of trachoma for most EAs was less than 20% and no EA is larger than 5,000 persons. The EAs are hereafter referred to as communities.

Forty-eight Gambian communities participated, located within four districts: Lower Baddibu, Central Baddibu, Foni Bintang Karanai, and Foni Kansala. Communities were eligible if they were located in the target district, community leadership gave consent to participate in the trial, and they had a prevalence of active trachoma greater than 5% in preschool age children, based on the best available data (no community had more than 50% trachoma). In The Gambia, a household is defined as persons who all eat together.

These communities varied in size between countries. In Tanzania, communities averaged around 1500 population. In The Gambia, communities' average population size was 700 persons.

### Data collection

Details on the PRET project methods, enrollment, and study procedures are described elsewhere [12] and summarized below.

In Tanzania, prior to mass treatment, trained research staff conducted a census of every household in the 32 communities. Baseline census information, the names, age, and gender of all persons residing in the household, was obtained from the head of household. Education completed by the head of household, distance to water, and presence of latrine were also collected for each household. These data were used to generate treatment books for mass distribution. In four randomly selected communities, all children less than ten years were screened at baseline for active trachoma (trachomatous follicular (TF) and/or trachomatous inflammation-intense (TI)) and ocular infection with *Chlamydia trachomatis*, using a commercially available test, Amplicor, which tests for the chlamydial plasmid (Roche Diagnostics, Indianapolis, IN). Data from this group were used to determine if non-compliance with mass treatment was associated with infection status at the baseline census.

In The Gambia, a census was completed in each of the 48 communities by trained research staff interviewing the head of the household. The data were used to generate treatment books to monitor mass treatment.

### Treatment

Mass treatment was a single dose of azithromycin, 20 mg/kg up to one gram. Azithromycin was offered to all members of the community aged over six months. For children younger than six months, tetracycline eye ointment was offered, and in The Gambia, pregnant women were also offered tetracycline eye ointment as an alternative. The regimen consisted of six weeks of tetracycline ointment, twice a day. Guardians of children less than six months were provided with tetracycline, and were responsible for complying with the regimen. In both countries, the minimum target for coverage of mass treatment was 80% of children aged less than ten years.

In Tanzania, a team of Community Treatment Assistants (CTAs) was trained in each community and assigned specific neighborhoods. They were given treatment books based on the census lists. The CTAs were responsible for providing treatment to all community residents, on pre-announced days. Azithromycin was offered at a central location in the neighborhood and, if necessary, at the household, was directly observed and noted in the treatment books. The CTAs were each provided a small incentive of 1000 TsH ($0.90) at the conclusion of mass treatment, if treatment verification showed that recorded treatment agreed with findings from the household treatment verification. Two to six CTAs were responsible for each community, and treatment days varied from two to five. Procedures identical to the National Program were carried out in Kongwa except that more supervision was provided and some communities were allowed additional days.

In The Gambia, a meeting was held with the community leaders to plan mass treatment for the community. There were typically one to two National Eye Care Programme (NECP) community ophthalmic nurses (CONs) in the community, and a variable number of ‘friends of the eye’ (nyateros) who were enlisted to help with distribution. Residents came to a central location within each community and treatment was directly observed. If any family member was missing, the family was asked to make sure the member came before the end of the day for treatment. The CON was notified in advance of persons who missed mass treatment, and asked to personally go and inform them of the subsequent treatment day in their community, and to advise treatment. One or two treatment days were offered. The drug distribution itself was provided by the NECP team, who received per diems of 200 dalasi/day ($7.88). This program was the NECP treatment for The Gambia.

### Statistical analyses

Coverage data was derived from treatment registers completed by the CTAs. For each community, we calculated the total coverage as the proportion of the censused population who had received either a tube of tetracycline or an observed dose of azithromycin. We calculated coverage of children as the proportion of children aged less than ten years who had received a dose of azithromycin or topical tetracycline; the denominator was all children aged less than ten years resident in the community during census. For each community, we then took households with children aged less than ten years and calculated the proportion of households where all children were treated, where no children were treated, and where some of the children were treated. This constitutes the observed proportions for each community.

Next, data were stratified according to community and 500 model simulations of household treatment distributions were run. These simulations assumed that children were treated and not treated at random, and for each community, the coverage of children less than ten years for the simulated data was set equal to the observed coverage. For each community, the observed proportions of households where all/none/some of the children were treated were compared with the simulated data. The intraclass correlation coefficient (ICC) of treatment status for children of the same household is reported, as a measure of clustering.

Because of the large variability of household size in The Gambia, we compared the ICC of households with fewer than four children to the ICC of households with four or more children.

To address the question if non-treatment was differential by infection status prior to treatment in Tanzania, logistic regression models were employed using treatment as the dependent variable and infection status prior to treatment as the predictor. We corrected standard errors to account for the clustering at household level using the generalized estimating equation approach.

## Results

Approximately 46,634 individuals, including 17,332 children under ten years, resided in the 32 Tanzanian communities at baseline. The mean community population in Tanzania was approximately 1,457 people. Community size ranged from 703–2496. The mean number of persons per household was similar across Tanzanian communities, and the average number of children aged under ten per household was 1.7 (Table 1).

**Table 1:** Baseline characteristics of communities by country.

	Tanzania	The Gambia
**Number of communities**	32	48
**Total population**	46634	33695
**Child population (under 10 years)**	17332	11321
**Average number of households across communities (SD)**	312.8 (87.0)	65.0 (20.9)
**Average household size across communities (SD)**	4.7 (0.32)	10.9 (2.0)
**Average population across communities (SD)**	1457.3 (429.0)	702.0 (243.1)
**Average child population across communities (SD)**	541.6 (180.1)	235.9 (86.0)

In The Gambia, 33,695 individuals, with 11,321 children below ten years, lived in the 48 Gambian communities (Table 1). The Gambia had an average community population of 702 people, with the size ranging from 327–1621. On average, there were approximately 11 individuals living in a Gambian household, and an average of four children aged less than ten years.

Coverage for both settings was high, reflecting the target of greater than 80% (Table 2). In Tanzania, the average coverage was 94% for children under age ten, ranging from 81%–100% in children 0–9 years and 65%–100% for all ages. The Gambian average coverage for children below age ten was lower, 89%, ranging from 62 to 99%. The range for total coverage of the population was 62 to 98%, with a mean coverage of 86%, similar to Tanzania.

**Table 2:** Average mass treatment coverage in communities by country.

	Tanzania	The Gambia
**Average community coverage of children (SD)**	94.0 (5.1)	88.7 (7.6)
**Average community coverage of total population (SD)**	88.7 (9.6)	86.2 (7.9)

**Figure 1:**
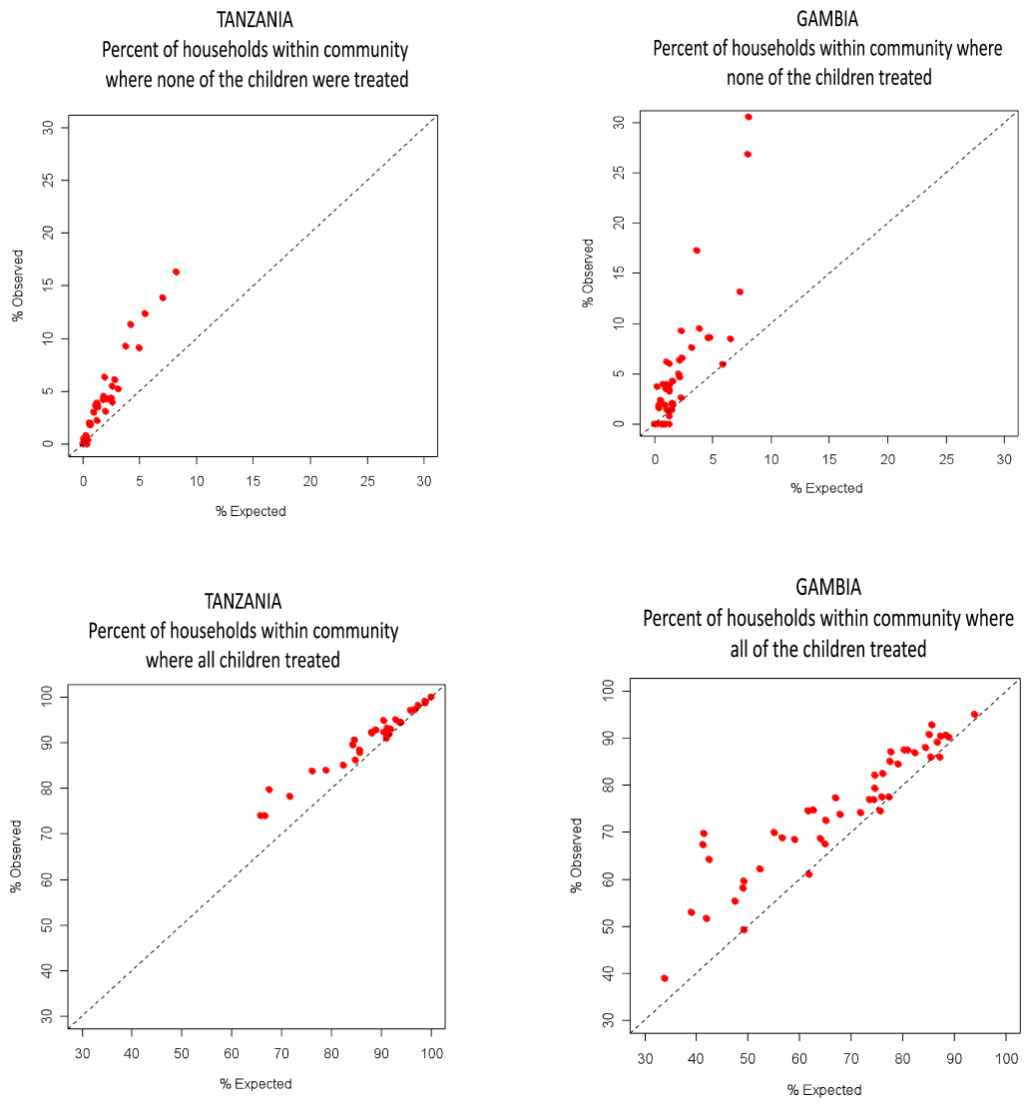
Comparison of expected vs. observed households with none/all children treated. The percentages of expected households were derived from simulations. Within each panel, each dot represents a community.

In both Tanzania and The Gambia, most households had all children treated (Table 3). The percentage of households where all children were treated, and the percentage of households where no child was treated, were both greater than would be predicted if non-treatment occurred at random, although the evidence for clustering was less strong in The Gambia (Figure 1).

**Table 3:** Household treatment status by country.

	Tanzania	The Gambia
**% of households where all children were treated**	89.8	75.2
**% of households where some children treated**	5.4	20.2
**% of households where none of the children were treated**	4.8	4.6

In Tanzania, the ICC for treatment status of children within households was 0.77 (95% CI 0.74–0.81). The mean percentage of households where no children under ten were treated was 4.6% (95% CI 3.2%–6.1%) compared to the estimate based on simulation with the presumption of non-treatment at random of 2.2% (95% CI 1.4%–2.9%).The mean percentage of households where all children were treated was 90.3% (95% CI 87.8%–92.8%), compared to the estimate based on simulation of 87.7% (95% CI 84.4%–90.1%). Thirty of 32 communities (94%) had greater than the predicted proportion of households with none of the children treated, if non-treatment was at random.

In The Gambia, the ICC was 0.55 (95%CI 0.51–0.59). On average, the percentage of households with no children treated was 4.8% (95% CI 3.0%–6.7%) compared to the estimate from simulations of 2.1% (95% CI1.5%–2.7%). The mean percentage of households with all children treated was 74.2% (95% CI 70.1%–78.3%) versus the estimate from simulations of 67.0% (95% CI 62.0%–72.1%). Of the 48 Gambian communities, 34 (70.8%) had significantly higher numbers of households with no children treated, compared to prediction if non–treatment was at random.

Because the range of household size was much greater in The Gambia, we compared the ICC for households with less than four children aged under ten years, to the ICC for households with four or more children in the Gambian data. For smaller household size, the ICC was 0.60 (95% CI 0.54–0.65), compared to Tanzania which was 0.77. The ICC was significantly lower for Gambian households with four or more children aged less than ten years, 0.50 (95% CI 0.45–0.56).

In the four Tanzanian communities where infection status was available for all children under ten years of age, households where none of the children were treated were no more likely to have children with infection than households where at least one child was treated (odds ratio 0.82 (95% CI 0.54–1.27). The PCR swab positive infection rate prior to treatment in children who were not treated was not statistically significantly different than that for children who were treated, 16.8% versus 23.9%, p = 0.09.

## Discussion

Our study in two different settings shows that in communities that carry out azithromycin mass treatment with high coverage, non-treatment (as well as all-treatment) of children clusters in households. There was strong evidence that treatment (and non-treatment) did not occur at random. Compared to assumptions of random non-treatment, we demonstrated significant levels of treatment and non-treatment clustering within households in Tanzania and The Gambia. A number of community programs for other diseases have also observed household clustering of treatment [13]–[15], and we have now found a similar trend for trachoma mass treatment.

Tanzanian households were more homogenous with respect to treatment compared to The Gambia. In part, this appears to be driven by the larger household size in The Gambia, where we observed higher ICC for households with less than four children compared to households with four or more children. With increasing numbers of children in a household, the bar is clearly higher for reaching “all children treated.” However, the ICC for Tanzania with four or fewer children was still greater than for The Gambia. This may also reflect differences in the approach to obtaining high coverage, and the higher coverage achieved in Tanzania. For children aged 0–9 years, Tanzanian communities had a 94% coverage compared to The Gambia's coverage of 89%. As coverage levels among children drop, it appears that the likelihood of more partially covered households increases.

Our data demonstrate that non-participation clusters, and that national programs should develop strategies to identify and treat households that do not participate, with examples of possible reasons for non-participation supported by the literature [16]–[18]. Our results support the argument that participation in public health programs is dependent on a number of social factors, which differ among households.

Our findings provide evidence that non-participation in mass drug administration is a non-random event; therefore, coverage surveys using cluster-sampling design should include a design effect to ensure that they have an appropriate sample size. If surveys estimate the number of persons treated, then an assumption that non-treatment is at random will result in sample sizes that are underpowered, and have the potential for erroneous estimates whose directionality is unpredictable. The design effect for Tanzania, for example, is 1.6 for an average household size of 1.8 children; because of the larger cluster size in The Gambia, the design effect is 2.7. A greater design effect means more household clusters must be included to achieve a minimum appropriate sample size.

In Tanzania, we also showed that prior to treatment, households with no treated children were no more likely to have at least one child with ocular *C. trachomatis* infection as households with some/all children treated. Consequently, households opting out of the treatment are not more likely to be infected, and thus do not represent a disproportionate threat to re-emergence within the community. If infection had been associated with households where none of the children was treated, even programs with high target coverage would have a more challenging job maintaining any reduction in trachoma prevalence. We also showed that infection rates prior to treatment were no different in children who were untreated compared to children who were treated, so while infected children still represent a threat of re-emergence, they are not differentially located in the untreated group. There was insufficient infection at baseline in The Gambia to address this question.

Although our study did not measure risk factors associated with participation, other community-based studies have shown that household factors such as absence of family notification [19], lack of family support for treatment [20], and increasing distance from the distribution site [21] were associated with non-participation. However, there is a need to investigate further the household factors related to clustering of non-treatment of children. Such research would help trachoma control programs by deepening their understanding of factors that might be altered to improve familial mass treatment participation.

We observed that where coverage in children was higher, the households tended to be more homogenous with respect to treatment. It may be easier for CTAs to encourage a guardian who has had some of their own children participate in mass treatment to treat the remaining family members, than to convince a guardian who has not brought any children for treatment to participate. In addition, with increasing number of days of mass treatment, it likely becomes easier to return to households a sufficient number of times to reach persons who may have been away. Consequently, as efforts persist to improve coverage, it may be that most households will have all members treated, or households will cluster with all non-treated members, and the community will consist of relatively fewer numbers of partially treated households.

There are some limitations to our analyses. In both countries, target coverage was very high, above 80%, thus limiting the generalizability of findings to communities where coverage rates are much lower; however, this is the coverage target that is recommended by WHO for trachoma control programs. We also constrained, for the simulations, the overall coverage in the community to match the observed coverage. Other approaches could have been used, such as taking the overall coverage for all communities combined. However, we wanted to assess household clustering, so using the overall community level value as the target for each community let us simulate treatment of children at random within the community. Finally, CTAs may have been tempted to report better than average coverage. However, treatment verification procedures reported excellent correspondence between CTA records and verification (fewer than 1% discrepancies).

In summary, we measured treatment clustering within households of countries with moderate and low trachoma infection, Tanzania and The Gambia, in the context of achieving high coverage rates. Participation in azithromycin mass treatment was not at random. Most communities had higher numbers of household with either all-treated or none-treated children. As treatment clustering is at the household level, an evaluation of household risk factors related to clustering may assist in understanding this phenomenon and contribute to the development of trachoma control programs with higher coverage. Regardless, national trachoma control programs need a plan to capture children who may be missed in mass treatment campaigns, and ensure continued participation through multiple rounds.

## Supporting Information

Checklist S1 STROBE checklist.
(0.08 MB DOC)

## References

[1] Cook JA (2008). Eliminating Blinding Trachoma.. N Engl J Med.

[2] Mariotti SP, Pascolini D, Rose-Nussbaumer J (2009). Trachoma: global magnitude of a preventable cause of blindness.. Br J Ophthalmol.

[3] Hotez PJ, Molyneux DH, Fenwick A, Kumaresan J, Sachs SE (2007). Control of neglected tropical diseases. N Engl J Med..

[4] Solomon AW, Zondervan M, Kuper H, Buchan JC, Mabey DCW (2006). Trachoma control: a guide for programme managers..

[5] West SK, Munoz B, Mkocha H, Holland MJ, Aguirre A (2005). Infection with Chlamydia trachomatis after mass treatment of a trachoma hyperendemic community in Tanzania: a longitudinal study.. Lancet.

[6] Katz J, Zeger SL, Tielsch JM (1988). Community and household clustering of xerophthalmia and trachoma.. Int J Epidemiol.

[7] Bailey R, Osmond C, Mabey DC, Whittle HC, Ward ME (1989). Analysis of the household distribution of trachoma in a Gambian community using a Monte Carlo simulation procedure.. Int J Epidemiol.

[8] West SK (1991). Epidemiology of trachoma in central Tanzania.. Int J of Epidemiology.

[9] Polack SR, Solomon AW, Alexander ND, Massae PA, Safari S (2005). The household distribution of trachoma in a Tanzanian community: an application of GIS to the study of trachoma.. Trans R Soc Trop Med Hyg.

[10] Broman AT, Shum K, Munoz B, Duncan DD, West SK (2006). Spatial Clustering of Ocular Chlamydial Infection over Time following Treatment, among Households in a Community in Tanzania.. IOVS.

[11] Blake IM, Burton MJ, Bailey JL, Solomon AW, West S (2009). Estimating Household and Community Transmission of Ocular Chlamydia trachomatis.. PLoS Negl Trop Dis.

[12] Stare D, Harding-Esch E, Munoz B, Bailey R, Mabey D (2010). Rationale and Design of Two Community-based Randomized Trials to Evaluate Mass Treatment with Azithromycin: The Partnership for Rapid Elimination of Trachoma (PRET).. Ophthalmic Epidemiology: in press.

[13] Rao MR, Naficy AB, Darwish MA, Darwish NM, Schisterman E (2002). Further evidence for association of hepatitis C infection with parenteral schistosomiasis treatment in Egypt.. BMC Infectious Diseases.

[14] Tallo VL, Carabin H, Alday PP, Balolong E, Olveda RM (2008). Is mass treatment the appropiate schistosomiasis elimination strategy?. Bulletin of the World Health Organization.

[15] La Vincente S, Kearns T, Connors C, Cameron S, Carapetis J (2009). Community Management of Endemic Scabies in Remote Aboriginal Communities of Northern Australia: Low Treatment Uptake and High Ongoing Acquisition.. PLoS Negl Trop Dis.

[16] Brieger WR, Otusanya SA, Oke GA, Oshiname FO, Adeniyi JD (2002). Factors associated with coverage in community directed treatment with ivermectin for onchocerciasis control in Oyo State, Nigeria.. Trop Med Int Health.

[17] Desmond N, Solomon AW, Massae PA, Lema N, Anemona A (2005). Acceptability of azithromycin for the control of trachoma in Norther Tanzania.. Trans R Soc Trop Med Hyg.

[18] Cromwell EA, Ngondi J, Gatpanb G, Becknell S, Kurd L (2009). Estimation of population coverage for antibiotic distribution for trachoma control: a comparison of methods.. International Health.

[19] Nuwaha F, Okware J, Ndyomugyenyi R (2005). Predictors of compliance with community-directed ivermectin treatment in Uganda: quantitative results.. Trop Med Int Health.

[20] Mathieu E, Lammie PJ, Radday J, Beach MJ, Streit T (2004). Factors associated with participation in campaign of mass treatment against lymphatic filariasis in Leogene Haiti.. Ann Trop Med Parasitol.

[21] Gardon J, Macé JM, Cadot E, Ogil C, Godin C (1996). Ivermectin-based control of onchoceriasis in northern Cameroon: Individual factors influencing participation in community treatment.. Transactions of the Royal Society of Tropical Medicine and Hygiene.

